# Psychometric properties of the Arabic version of PHEEM applied on a sample of medical residents in Syria

**DOI:** 10.1186/s12909-024-05731-5

**Published:** 2024-07-05

**Authors:** Ghaith Alfakhry, Rama Kodmani, Imad Addin Almasri

**Affiliations:** 1 Education Quality and Scientific Research Office, Al-Sham Private University, Damascus, Damascus Governorate, Syria; 2 Department of Education, University of Oxford, 15 Norham Gardens, Oxford, OX2 6PY UK; 3 University Hospital of Dermatology and Venereology, Damascus University, Damascus, Damascus Governorate Syria; 4 Department of Applied Statistics, Faculty of Economics, Damascus University, Damascus, Syria; 5 Stemosis for Scientific Research, Damascus, Syria

**Keywords:** Psychometric analysis, Validation, Reliability, Arabic language, Postgraduate Hospital Educational Environment measure, PHEEM, Clinical learning environment, Syria

## Abstract

**Background:**

The clinical learning environment (CLE) plays a crucial role in shaping the learning experiences and professional development of medical professionals. Understanding and optimising this environment is essential for improving doctors’ knowledge acquisition, clinical skills, and overall well-being. The development of the Postgraduate Hospital Educational Environment Measure (PHEEM) and its translation to numerous languages has been a milestone in clinical education. Even though PHEEM was recently translated into Arabic, its psychometric properties in this form remain unevaluated. Therefore, this study aims to conduct a comprehensive psychometric analysis of the Arabic version of the PHEEM questionnaire.

**Methods:**

This is a cross-sectional questionnaire survey validation study. The defined population were medical residents in Damascus, Syria. A paper-based survey as well as an online-based one were conducted using several non-probability sampling methods namely, convenience, river and, snowball sampling between June 15, 2023, and June 21, 2023. Both exploratory (EFA) and confirmatory (CFA) factor analyses were conducted. Several psychometric criteria were applied including scree plot, eigenvalue > 1.5 and the ‘proportion of variance accounted for’ criterion.

**Results:**

A total of 543 participants completed the questionnaire (56.9% female). Kaiser-Meyer-Olkin measure for sample adequacy was high (0.937) and the *P*-value for Bartlett’s test was < 0.001. EFA revealed five meaningful factors which were labelled: *perception of teachers*,* learner’s engagement and social participation*,* external regulation*,* work culture*,* and living conditions.* These factors had the following eigenvalues: 12.6, 2.18, 2.03, 1.86, and 1.41 respectively, with a total explained variance of 43.45%. Cronbach’s Alpha was 0.938. CFA confirmed the model structure of EFA (SRMR = 0.067 and RMSEA = 0.066). The Average Variance Explained (AVE) value of any given factor was > 0.7.

**Discussion:**

The Arabic PHEEM inventory demonstrated satisfactory psychometric properties. The extracted domains are of theoretical relevance to the psychosocial-material conceptual framework for learning environment. Nonetheless, this validation was performed in the Syrian context; therefore, future studies in other Arabic countries are recommended to support the applicability of Arabic PHEEM in the wide Arab World.

## Introduction

The learning environment (LE) is fundamental in shaping and nurturing a learner’s character and skills. LE can be either conducive or disadvantageous to learning, and the effects of both have been discussed extensively in the literature [1–3]. In spite of the various proposed interventions to address the deficiencies in the LE, researchers and curriculum designers still face difficulty in the conceptualization and measurement of the LE, especially in complex changing contexts such as in clinical education where learning is intertwined with clinical practice [2, 4].

Academics and theorists argued that the LE perimeter extends beyond a single educational theory and is rather conceptualized in a complex psycho-socio-physical construct where multiple theories can be applied [5]; to name a few: ecological psychology [6], situated cognition [7], activity theory [8], workplace learning [9] and socio-materiality [10, 11]. The conceptual framework adopted in this current study was introduced by Gruppen et al. in 2019 [5]: this framework has two inseparable dimensions: the psychosocial dimension and the material dimension. The psychosocial one is comprised of three components: the personal, the social and the organizational; the material dimension, on the other hand, has two components: the physical and virtual spaces.

In 2005, Roff et al. developed the Postgraduate Hospital Educational Environment Measure (PHEEM) to quantitatively assess the clinical learning environment for postgraduate medical training programs, and ever since, it has become one of the most widely used instruments globally [12, 13]. The PHEEM has been developed using a combination of grounded theory and the Delphi process [12] and has been identified as a highly reliable, valid and practical instrument [13]. This inventory is comprised of 40 items which were claimed to measure three distinct domains, namely: autonomy; teaching; and social support [12]. These domains were qualitatively validated [12] at that time and have not been completely supported by psychometric analysis which showed conflicting results and various structures and models. For example, three studies posited that the PHEEM questionnaire measures a single principal dimension [14–16]. Another study, in which the Dutch version of PHEEM was used, suggested a three-dimension structure comprised of *learning content and coaching*, *work culture*, and *external regulation* [17]; all of which corresponded with educational theories. Other studies also supported the multidimensionality of the instrument [18, 19]. The variety in factor structure points to the sophistication, complexity and correlated constructs of the LE [13].

The PHEEM has been translated and validated into several languages including Spanish, Danish, Greek, Japanese, and Persian, and most recently has been translated into Arabic [19–25]. The Arabic version of PHEEM was checked for both face and content validity [24]. Notwithstanding, psychometric analysis has not been conducted to establish its reliability, homogeneity, construct validity, factor structure, and model fit of the tool. Additionally, it has been recommended that PHEEM studies should conduct their own independent factor analysis to confirm the appropriate factor structure for their respective setting [13]. The application of PHEEM in the Arabic World has not been as popular as the DREEM inventory which has a published Arabic version [26]. Questionnaires available in the mother tongue of the target population can arguably be more accessible and preferred to the English version even when the target population is proficient in English. This is demonstrated well in a PHEEM study conducted in Saudi Arabic where almost all participants (97%) preferred to fill in the Arabic version of PHEEM over the English one [27]. The validation of the Arabic PHEEM would provide curriculum developers in Syria and the Arab World with the proper tools to navigate issues in the clinical learning environment and propose appropriate interventions and reforms that could foster a conducive learning environment for resident doctors. The benefits of using PHEEM are well-illustrated in the literature [13]. Thus, this study set out to investigate the psychometric properties of the developed Arabic PHEEM inventory on a sample of medical residents located in Damascus, Syria to see if it is appropriate for assessing the clinical learning environment in this context. The first psychometric property is the validity of the questionnaire, and to validate a questionnaire is to find evidence to support the fact that the instrument is measuring what it is supposed to measure. The second property is the reliability of the questionnaire, which is defined as the reproducibility or consistency of scores among raters.

## Materials & methods

This is a cross-sectional study with a quantitative positivist theoretical orientation. The study was approved by the Ethics Committee of Al-Sham Private University (no. 51,655). Informed consent was sought from respondents whose participation was anonymous.

### Participants and settings

Medical education training in Syria is comprised of six years of undergraduate training and three to seven years of postgraduate training depending on the speciality. Postgraduate residency training is run mainly by two governmental institutes, the Ministry of Higher Education and the Ministry of Health and each institute deploys its resident doctors in separate hospitals or healthcare centers. There are other postgraduate medical training programs run by the Ministry of Defense, Ministry of Internal Affairs and the Department of Police. Nevertheless, the majority of resident doctors in Syria are enlisted either by the Ministry of Health or the Ministry of Higher Education. For the purpose of validating the newly translated and cross-validated Arabic PHEEM, responses were collected from a sample of medical residents from hospitals located in Damascus, Syria. Multiple specialities were included in the study. The literature shows that at least ten subjects per item are necessary to conduct item analysis and exploratory analysis [28–30]; hence, for a questionnaire containing 40 items, a sample size of 400 (40*10) will be required. To conduct confirmatory factor analysis or to conduct power analysis, the recommendation is to use 300–500 subjects [31, 32].

### Data collection

The survey was conducted between June 15, 2023 and June 21, 2023. Due to a lack of accurate good record-keeping, an accurate sampling frame could not be established. Henceforth, applying the probability sampling technique was not possible. Given these conditions, an alternative feasible sampling method that was judged appropriate is to use the wide reaching connections the researchers had with residents across teaching hospitals in Damascus.

River sampling which is an online sampling technique [33] was used where the Arabic PHEEM was developed electronically using Google Forms and later posted on social media groups dedicated to the targeted medical residents. Convenience and snowball sampling using a paper-based survey was also utilised in conjunction with the online survey to reach the recommended sample size. Participants completed the Arabic PHEEM voluntarily and anonymously from several hospitals in Damascus Governorate, mainly in nine major hospitals which were: Obstetrics Hospital, Dermatology Hospital, Children’s Hospital, Almousat Hospital, Al-Assad Hospital, Ophthalmology Hospital, Ibn Alnafees Hospital, Red Crescent Hospital and Al-Mojtahed Hospital. Demographic information that was collected included age, sex, specialization, hospital, and year of study.

### Postgraduate Hospital Educational Environment measure (PHEEM)

PHEEM is originally a 40-item questionnaire answered on a five-point Likert scale (0 = strongly disagree, 4 = strongly agree). The questionnaire has 36 positively phrased items and 4 negative ones which were scored in reverse so that the higher the score the better the perception is. In this study, the linguistically validated Arabic version of the PHEEM developed by Alfakhry et al. (2024) was used [24]. Items no. 7, 11, 17 differ in translation from the original version. For full details on the translated Arabic version of PHEEM, please see Alfakhry et al. (2024) study [34]. The translation process was rigorous and followed the guidelines of Sousa and Rojjanasrirat [35] for translation, validation and cross-cultural adaptation of instruments.

### Psychometric analysis

Content validity was addressed when the questionnaire was translated in the previous study [24]. The recommendations of Schönrock-Adema et al. [17] for factor analysis and validation of instruments were followed. Before conducting factor analysis, data normality was checked using skewness and kurtosis as indicators. Because of the large sample size [36] and the normal distribution of scores, parametric statistics were applied [37, 38].

The internal consistency of the instrument was measured using Cronbach’s Alpha along with Guttman’s Lambda test and split-half reliability method. Cronbach’s Alpha value within the range of (0.7–0.8) is interpreted as acceptable, between (0.8–0.9) are good, and scores > 0.9 are considered excellent. Split-half reliability measures the internal consistency by calculating the correlation between the scores of two halves of the scale to see how consistently the items measure the same construct. The Guttman’s Lambda test provides six measures of reliability based on the split-half method.

To ensure that the sample size is appropriate for conducting factor analysis, Kaiser-Meyer-Olkin (KMO) measure for sampling adequacy and Barlett’s test of sphericity was conducted. In the KMO test, values closer to 1 indicate that the data is appropriate for applying factor analysis; a value over 0.8 is recommended [39]. As for Barlett’s test, the significance value must be less than 0.05 for the factor analysis to be acceptable.

### Exploratory Factor Analysis (EFA)

Principal Axis Factoring was conducted with Promax rotation and Kaiser Normalization to investigate the internal structure of the Arabic PHEEM. Three criteria were applied to determine the number of factors (components) to be retained [17]: (1) point of inflexion on the scree plot (Cattell criterion); (2) Eigenvalues > 1.5; (3) ‘Proportion of variance accounted for’ minimally explained about 5% of the variance. It is worth noting that a cut-off point is recommended to be decided by the Cattell criterion (inflexion point) when there are more than 30 variables in the dataset [40, 41].

To evaluate the theoretical sensibility of the best solutions, Hatcher’s interpretability criteria [42] were adopted. The interpretability criteria are: (4a) a given component has at least three variables with significant loadings > 0.40 (4b) variables loading on the same component measure the same construct; (4c) variables loading on different components measure different constructs; (4d) the rotated factor pattern demonstrates simple structure which is: (i) most variables load high on only one component and low on the other components; (ii) most components have high factor loadings for some variables and low loadings for the rest.

To ascertain the interpretability criteria (4b and 4c), two medical education experts interpreted the factors and reached a consensus on the interpretation of factors and the best factor solution.

### Confirmatory Factor Analysis (CFA)

Using SPSS AMOS, CFA was conducted to verify the model yielded from the principal axis factoring in the EFA. Whereas EFA focuses on reducing the data into factors that make sense, the CFA is used to validate and evaluate the model fitness generated in the EFA.

The potent constructs revealed in the EFA were evaluated for fit of Structural Equation Models using absolute fit indices which were the Root Mean Square Error of Approximation (RMSEA) and Standardized Root Mean Square Residual (SRMR). RMSEA values ≤ 0.05 are indicative of a very good fit, 0.05 ≤ RMSEA ≤ 0.08 a fair fit, and values larger than 0.10 show a poor fit [43, 44]. The threshold for SRMR for acceptable model fit is SRMR ≤ 0.08 [45]. The Average Variance Extracted (AVE) was used to assess convergent validity; AVE must be ≥ 0.5 [46]. Correlation between factors (sub-scales) was measured to assess discriminant validity. The weaker the correlation, the higher the discriminant validity is [47]. The Critical Ratio (CR) method was used to examine the discrimination of scale items. If the CR is less than 3.0 (*P* > 0.05), discrimination of the item is poor and thus it is deleted [48].

Google Forms was used as the online platform to conduct the online survey. Data processing and analysis were conducted using Microsoft Excel (2019), IBM SPSS Statistics for Windows, version 26 (IBM Corp., Armonk, N.Y., USA) and SPSS AMOS 24.

## Results

The questionnaire was completed by 543 medical residents from 34 different departments in nine major hospitals located in Damascus; 312 participants were recruited from the paper-based survey and 231 were from the online survey. The percentage of female participants was 56.9% (*n* = 309) and the age mean was 26.3 (SD = 2.0). Response rates for each item were between 98.0% and 100%.

### Exploratory factor analysis

**Fig. 1:**
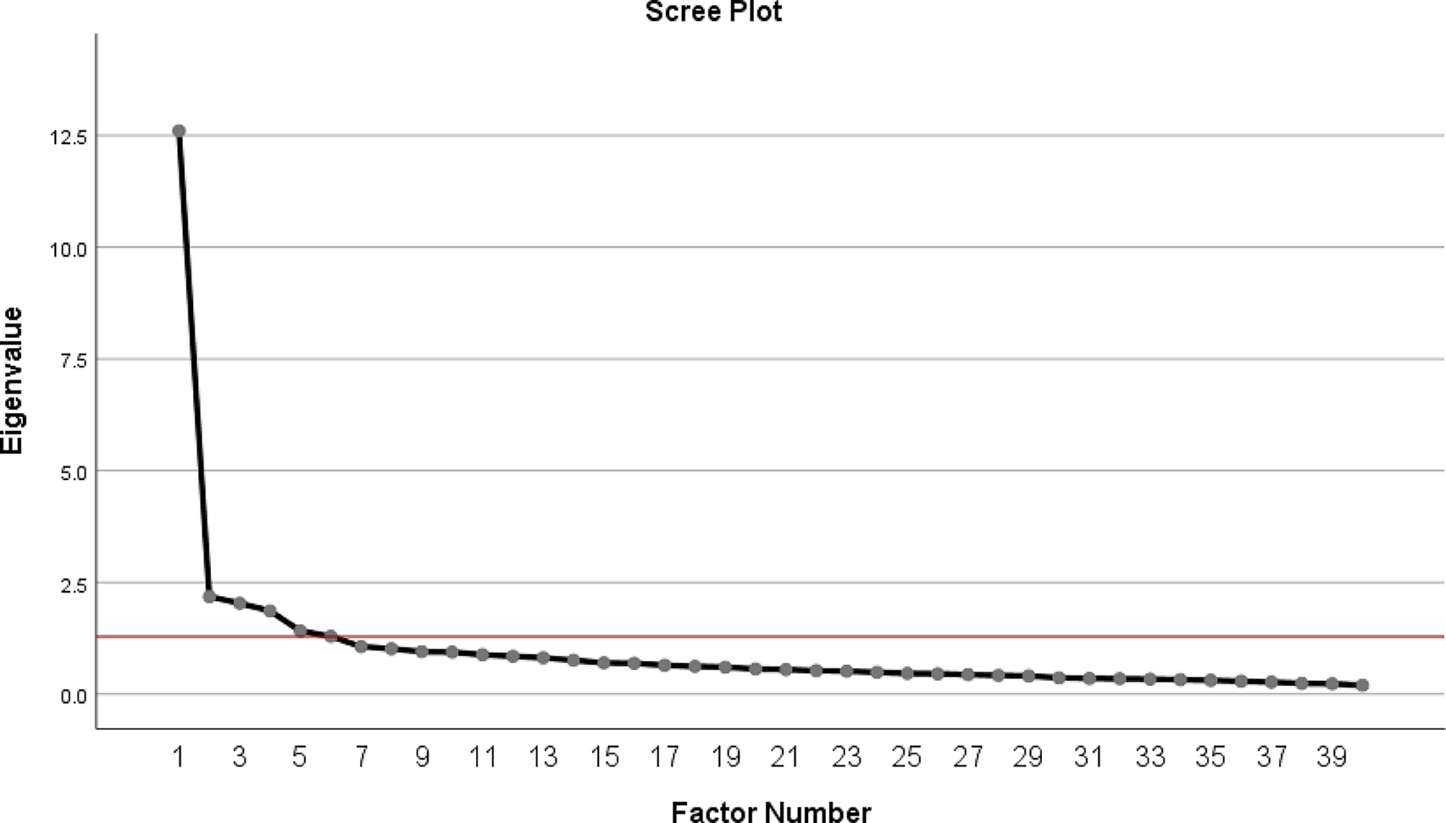
Scree plot of the eigenvalues of the factors

KMO’s test value was 0.937 and the *P*-value for Bartlett’s test was < 0.001; therefore, the sample size was deemed adequate for conducting factor analysis. The scree plot showed a sharp point of inflexion after the first factor (Fig. 1). Only four factors (components) had eigenvalue > 1.5 (criterion 2), with values ranging between 1.86 and 12.60; the eigenvalue of the fifth factor was 1.41. Of these factors, each of the first four factors accounted for more than or approximately 5% of the variance (criterion 3) independently. The fifth factor explained 3.53% of the variance (Table 1).

**Table 1:** Factor analysis components and their initial eigenvalues

Component	No. of items	Eigenvalues	% of Variance	% of Cumulative
1	10	12.60	31.50	31.50
2	13	2.18	5.45	36.96
3	5	2.03	5.08	42.04
4	6	1.86	4.65	46.70
5	2	1.41	3.53	50.23

Taken into consideration the eigenvalue and the proportion of the variance explained, two solutions were considered, the 4- and 5-factor solutions. All four factors still contained at least three variables with loadings over 0.40 (criterion 4a). The fifth factor contained two variables with significant loadings, 0.63 and 0.71 (Table 2). The inflexion point of the curve was observed between factors 4 and 5 (Fig. 1).

**Table 2:** Factor loadings of the final 5-factor solution

		Component				
No.	Item	1	2	3	4	5
35	My clinical teachers have good mentoring skills	0.926				
28	My clinical teachers have good teaching skills	0.791				
31	My clinical teachers are accessible	0.759				
15	My clinical teachers are enthusiastic	0.705				
10	My clinical teachers have good communication skills	0.658				
23	My clinical teachers are well organized	0.623				
39	The clinical teachers provide me with good feedback on my strengths and weaknesses.	0.606				
40	My clinical teachers promote an atmosphere of mutual respect	0.555				
19	I have suitability access to career advice	0.484				
21	There is access to an educational programme relevant to my needs.	0.320				
18	I have the opportunity to provide continuity of care					
37	My clinical teachers encourage me to be an independent learner					
30	I have opportunities to acquire the appropriate practical procedures for my grade		0.655			
27	I have enough clinical learning opportunities for my needs		0.599			
22	I get regular feedback from seniors		0.593			
29	I feel part of a team working here		0.561			
7	There is discrimination based on (Ethnicity, religion, socioeconomic status, age) in this post		0.532			
16	I have good collaboration with other doctors in my grade		0.517			
13	There is sex discrimination in this post		0.459			
6	I have good clinical supervision at all times		0.441			
34	The training in this post makes me feel ready to be a SpR/Consultant		0.437			
5	I have the appropriate level of responsibility in this post		0.422			
36	I get a lot of enjoyment out of my present job		0.367			
33	Senior staff utilize learning opportunities effectively		0.351			
12	I am able to participate actively in educational events		0.324			
3	I have protected educational time in this post			0.817		
2	My clinical teachers set clear expectations			0.684		
9	There is an informative Junior Doctors handbook			0.623		
4	I had an informative induction programme			0.620		
1	I have a contract of employment that provides information about hours of work			0.516		
14	There are clear clinical protocols in this post					
38	There are good counselling opportunities for junior doctors who fail to complete their training satisfactorily					
32	My workload in this job is fine				0.596	
17	My work hours are acceptable				0.587	
8	I have to perform inappropriate tasks				0.571	
25	There is a no-blame culture in this post				0.480	
11	I am summoned for unnecessary matters when I am on call				0.455	
24	I feel physically safe within the hospital environment				0.346	
20	This hospital has good quality accommodation for junior doctors, especially when on call					0.713
26	There are adequate catering facilities when I am on call					0.638

“Extraction Method: Principal Axis Factoring. Rotation Method: Promax with Kaiser Normalization.”

a. Rotation converged in 15 iterations.

Even though the minimum number of items for any factor is set to be three, expert opinion supported the meaningfulness and importance of factor 5; therefore, the 5-factor solution was maintained; future studies may want to add items relevant to this factor to further support its addition as a factor. The 5-factor solution explained 43.45% of the variance cumulatively. The first factor contained items related to how residents perceive their clinical teachers and therefore, it was interpreted by the experts as the *perception of teachers.* The second factor contained items relating to the social atmosphere and engagement in learning activities and was interpreted as the *learner’s engagement and social participation.* The third factor focused mainly on the rules and policies of the hospital, and it was labelled as *external regulation*. The fourth factor contained five items concerning being assigned appropriate tasks, working hours and workload, “no-blame” culture and being safe; experts labelled the fourth factor as *work culture.* The fifth component contained two items about accommodation and catering facilities, and it was identified by experts as *living conditions*. Four items, namely no. 14, 18, 37 and 38 did not load on any of the five factors and therefore were excluded from the analysis.

### Reliability of the 36-item instrument

Cronbach’s Alpha for the 36-item scale was 0.938. As for the Cronbach’s Alpha of the first four factors, they were 0.914, 0.830, 0.775 and 0.772 respectively; the fifth subscale which had only two items had a Cronbach’s Alpha of 0.67.

Split-half reliability statistics showed that the correlation between the first half of the 36 items and the second half was 0.80, and the Spearman-Brown coefficient value was 0.89.

Guttman’s Lambda- 1, 2, 3, 4, 5 and 6 scores were all high (≥ 0.89): Lambda-2, 3 and 6 which are similar to Cronbach’s Alpha are over 0.94, Lambda-4 which is the Guttman split-half reliability is 0.89 and Lambda-6 is 0.95. All six Lambda’s indicate high reliability of the 36-item instrument.

### Confirmatory Factor Analysis (CFA)

**Fig. 2:**
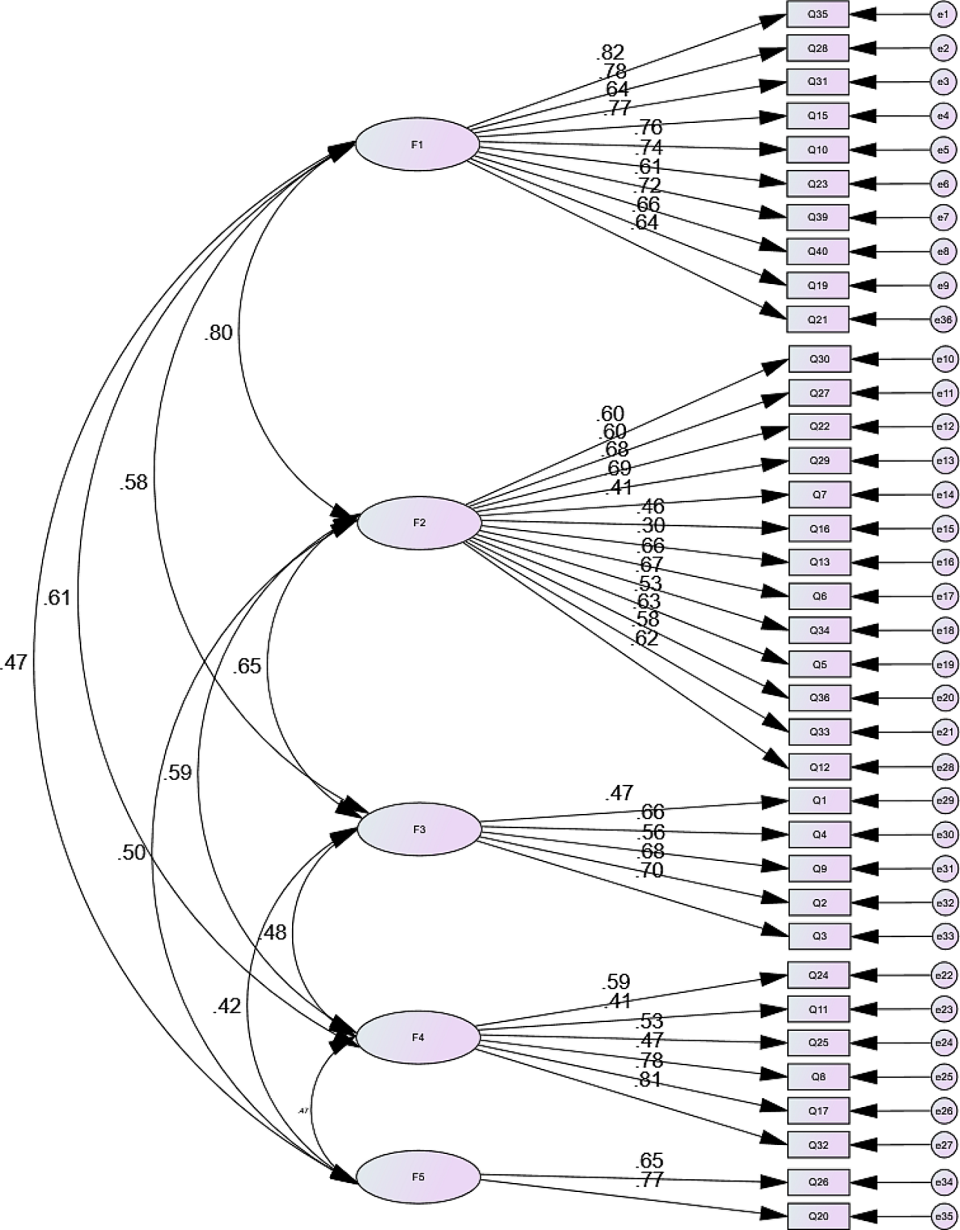
Path diagram of the five factors and CFA factor loadings

The CFA confirmed the results of the exploratory factor analysis, supporting the five-factor structure of the Arabic version of PHEEM; the results of the absolute fit indices were SRMR = 0.067 and RMSEA = 0.066 (90% Confidence interval (0.063–0.069)). A total of 32 items out of 36 items loaded significantly (*P* < 0.001) on the proposed 5-factor solution that we revealed using the Principal Axis Factoring. The four items that did not show significant factor loadings with their proposed factors are items no. 35 (factor: 1), 30 (Factor:2), 12 (factor:3), and 24 (Factor:4). CFA factor loadings along with standard error and critical value of each item with its pre-specified factor are shown in Table 3; standardized regression weights (factor loadings) ranged between 0.405 and 0.815 apart from item no. 13 whose loading was 0.299. The AVE values were all above 0.7. A graphical representation of the CFA model with factor loadings are shown in **Fig. 2;** factors are shown in oval shapes; factor loadings on each variable are also indicated. The factor correlation matrix presented in Table 4 shows that r values between each two factors ranged between 0.32 and 0.62.

**Table 3:** Factor loadings (regression coefficients) for the pre-specified model as suggested by EFA

Factor	Item	Estimate	S.E.	C.*R*.	*P*	AVE
F1	35	0.823				0.916
	28	0.778	0.049	20.679	< 0.001	
	31	0.639	0.054	15.971	< 0.001	
	15	0.773	0.052	20.496	< 0.001	
	10	0.757	0.049	19.877	< 0.001	
	23	0.745	0.050	19.436	< 0.001	
	39	0.611	0.055	15.041	< 0.001	
	40	0.718	0.053	18.551	< 0.001	
	19	0.66	0.056	16.559	< 0.001	
	21	0.639	0.052	15.908	< 0.001	
F2	30	0.615				0.793
	27	0.617	0.095	11.947	< 0.001	
	22	0.682	0.094	12.924	< 0.001	
	29	0.693	0.084	13.085	< 0.001	
	7	0.405	0.093	8.391	< 0.001	
	16	0.461	0.067	9.417	< 0.001	
	13	0.299	0.165	6.340	< 0.001	
	6	0.655	0.098	12.538	< 0.001	
	34	0.68	0.089	12.902	< 0.001	
	5	0.525	0.095	10.542	< 0.001	
	36	0.629	0.097	12.171	< 0.001	
	33	0.58	0.086	11.400	< 0.001	
F3	12	0.646				0.831
	1	0.446	0.095	8.900	< 0.001	
	4	0.648	0.098	12.184	< 0.001	
	9	0.541	0.077	10.465	< 0.001	
	2	0.661	0.085	12.360	< 0.001	
	3	0.683	0.099	12.670	< 0.001	
F4	24	0.584				0.812
	11	0.412	0.090	8.080	< 0.001	
	25	0.53	0.095	9.905	< 0.001	
	8	0.465	0.100	8.932	< 0.001	
	17	0.786	0.126	12.877	< 0.001	
	32	0.814	0.118	13.074	< 0.001	
F5	26	0.658				0.885
	20	0.766	0.132	8.790	< 0.001	

Estimate: factor loadings. S.E. standard error, C.R.: critical ratio

**Table 4:** Correlation matrix for each of the five factors

Factor	F1	F2	F3	F4	F5
**F1**	1				
**F2**	0.62	1			
**F3**	0.56	0.55	1		
**F4**	0.46	0.40	0.42	1	
**F5**	0.48	0.40	0.49	0.32	1

## Discussion

This study was set out to explore the psychometric properties of the Arabic version of PHEEM. To this end, the Arabic PHEEM was applied to a sample of medical residents working at hospitals in Damascus, Syria. KMO test for the adequacy of sampling gave a very high value (0.937) which means that the factor analysis should show distinct and reliable factors [40]. Thereafter, exploratory and confirmatory factor analyses were conducted. Results of the exploratory factor analysis revealed two possible solutions: the 4- and 5-factor solution. Despite that factor five has only two variables and an eigenvalue slightly under 1.5 (1.41), it was not omitted due to the meaningfulness and importance of the items as per experts’ recommendations. EFA reduced the number of items to 36 which were approved by the experts. The five-factor solution explained 43.45% of the variance. Experts labelled the five factors as follows: *perception of teachers*,* learner’s engagement and social participation*,* external regulation*,* work culture*, and *living conditions* respectively. These labels are based on previous studies that showed similar factor structure [19]; factors three and four’s labels were taken from a previous study [17]. The model suggested by EFA was confirmed using CFA; results showed that the model fitted fairly and explained the majority of items, RMSEA value indicated a fair fit and the SRMR value was acceptable and well below the acceptable threshold (≤ 0.08). AVE values were ≥ 0.5 and this establishes the convergent validity of the instrument [46]. In addition, the correlation matrix showed good discriminant validity of the factors as the correlation was weak [47]. Four items (no. 12, 24, 30, 35) did not load significantly; however, due to their conceptual importance, they were retained. All reliability indicators and coefficients supported the high internal consistency of the instrument.

The five factors extracted in the current study correspond well with the conceptual framework of the learning environment described by Gruppen et al [5] which describes two dimensions (psychosocial and material) and five components, the personal, the social, the organizational, the physical spaces and virtual spaces component: The first and most important factor that explains the majority of the variance, *perception of teachers*, corresponds well with the social component of faculty/learner interactions (mentoring, communication, feedback, trust) [5]; unlike previous studies [12, 13] which named this factor *perception of teaching*, our experts pointed out that the statements included do not evaluate the teaching act itself but rather medical teachers, their characteristics (accessible, enthusiastic), and teaching skills. Therefore, the label “*perception of teachers*” was preferred. The second factor, *learner’s engagement and social participation* corresponds both with the personal component (interest, engagement and emerging autonomy) and social component (becoming part of a team, interactions for learning) [5]; the third factor, *external regulation*, is inspired from Schönrock-Adema et al. study in their factor analysis of PHEEM [17]-this factor can be associated with the *organizational component*. The fourth factor, *work culture*, can also be linked to the organizational component namely, organizational culture and practices [5]. The fifth factor is comprised of two items related to catering and accommodation, which we decided to name: *living condition* and it corresponds with the physical spaces in Gruppen et al. framework [5]. In this manner, our factor structure becomes theoretically sensible. In fact, a study in 2009 reported a similar 5-factor structure solution of PHEEM which further supports our findings [19]; in both studies, factors 1, 4 and 5 have approximately the same items [19].

Our study supported the notion that PHEEM is a multi-dimensional instrument rather than a uni-dimensional one unlike what Boor et al [14] proposed. Additionally, our analysis does not support the three-domain structure of PHEEM as proposed by the original study [12]. Determining the number of factors largely depends on deciding the cut-off point; some adopt the Kaiser-Guttman criterion of including all factors with eigenvalue > 1; others use the Cattel criterion explaining that it is more suitable because the PHEEM includes over 30 items [40]. Boor et al. who found that PHEEM was unidimensional adopted a stricter eigenvalue of 2.1 and discarded all factors below [14]. There are two studies [17, 18] that somewhat concurred with the original 3-factor solution as proposed in the original study by Roff et al [12, 17, 18]; however, Schönrock-Adema et al. named them differently [17]. Similar to our study, there are two studies reporting five factors for PHEEM [19, 23]; nonetheless, neither of these studies explained their factors theoretically within an accepted conceptual framework of the LE.

Another aspect that should be considered when comparing our factor structure to the original 3-subscale structure [12] is that our study utilized a quantitative positivist mathematical and statistical approach whereas the original study used qualitative interpretivist approaches (grounded theory, focus groups, nominal group, Delphi technique) [12]. While some argued that the original structure cannot be proven by a different epistemological approach [49], one study found some degree of agreement with the original study structure [12]. In our study, Factors one, two and three (*perception of teaching*,* learner’s engagement and social participation and work culture*) closely resemble the three subscales (autonomy, teaching and social support) suggested in the original study. The specific cultural variables and values and unique interpretations of the instrument by our participants in the Syrian setting which differ from the context of many PHEEM studies might have also played a role in determining the number of factors.

The factor solution revealed in this study explained 43.45% of the variance which is considerably higher than the variance explained by studies adopting the one-factor solution whose explained variance was 31% [14], 32.8% [16] and 19.7% [15]. In comparison, Schönrock-Adema et study gave a 3-factor solution that explained 37.7% of the variance. Whereas Wall et al. 3-factor solution had an explained variance equal to our study (43%) [18]. In comparison to our study, other studies that gave a five-factor solution had higher explained variance (52.8% [23] and 58% [19]). Our findings suggest that Factor 1 which contains items related to teaching is the most important subscale of the questionnaire and that is concurrent with the Chilean PHEEM study [19]. Cronbach’s Alpha of our solution shows high internal consistency (0.938) and each of our five subscales showed excellent to acceptable reliability; our fifth factor (items 20 and 26) showed higher reliability (Cronbach’s Alpha = 0.67) than its counterpart fifth factor in the Riquelme et al. study (Cronbach’s Alpha = 0.426) which also had the same two items [19]. A systematic review of PHEEM studies showed that Cronbach’s Alpha values of the instrument ranged from 0.84 to 0.989 [13]. However, unlike the majority of previous studies that used only a single indicator of reliability (Cronbach’s Alpha), our study calculated the reliability of the instrument using three different indicators (Cronbach’s Alpha, Split-half, Guttman).

There are some limitations that need to be considered in our study. First, although the current study provided validity evidence of the Arabic PHEEM, it only achieved that in the Syrian context. There are 22 Arabic countries that might have different socio-cultural-organizational settings. Thus, and in line with Chan et al. suggestions [13], researchers adopting our Arabic PHEEM should consider performing an independent factor analysis to identify the optimal factor structure in their respective contexts. Given the cultural and contextual similarities between Syria and other countries in the region, the validity evidence established for the Arabic PHEEM questionnaire in this study may also be applicable in those contexts. The sampling methods (convenience, river, and snowball) used in this study are another shortcoming of the study as these methods are not optimal for factor analysis [50]; nonetheless, the very large sample size (*n* = 543) collected from different teaching hospitals and the high Kaiser-Meyer-Olkin index support our sample adequacy which in turn support the output of our factor analysis. Another limitation of this study is the heterogeneity of our sample, which included residents from various departments and hospitals. Ideally, a multi-level confirmatory factor analysis (CFA) would have been performed to address the sample structure. However, given that many residents in Damascus rotate across multiple hospitals, our sample can somewhat be treated as a single level; thus, due to the difficulty of sorting the majority of participants into particular hospitals, the sample was treated as one-level. Readers should interpret our findings with these limitations in mind.

Now that the validity and reliability of the Arabic version of PHEEM are established, educators, curriculum developers and accreditors in Arabic countries will be better equipped to gauge the clinical learning environment at teaching hospitals, identify areas of weaknesses and design appropriate interventions that could ensure a conducive clinical learning environment that optimizes both working and learning [51].

## Conclusion

The Arabic version of PHEEM exhibited satisfactory psychometric properties in terms of both validity and reliability and is suitable for the measurement of the clinical learning environment at teaching hospitals from the perception of medical residents, though adjacent independent factor analysis is advisable for future PHEEM studies conducted in other Arabic countries in order to support PHEEM’s applicability in the Arabic World.

## Data Availability

The datasets used and/or analysed during the current study are available from the corresponding author upon reasonable request.
